# Changes in perceived health status in older men and women during the COVID-19 pandemic

**DOI:** 10.1093/eurpub/ckac130.017

**Published:** 2022-10-25

**Authors:** G Autric-Tamayo, M Sánchez-Román, C Rodríguez-Blázquez, JM Rojo-Abuín, A Ayala, MJ Forjaz, MA Molina, F Rojo-Pérez, V Rodríguez-Rodríguez, G Fernández-Mayoralas

**Affiliations:** 1 Centro Nacional de Epidemiología, Instituto de Salud Carlos III, Madrid, Spain; 2 Grupo de Investigación en Envejecimiento, Instituto de Economía, Geografía y Demografía CSIC, Madrid, Spain; 3 Unidad de Análisis Estadístico, UAE-CCHS, CSIC, Madrid, Spain; 4 Universidad Carlos III, Madrid, Spain; 5 UNED, Madrid, Spain

## Abstract

**Background:**

The COVID-19 pandemic has severely impacted older people. The disease and the measures to combat it have had a differential impact according to gender, with higher mortality rates in men and worse psychological and social consequences in women. The objective of this work is to analyze the changes in perceived health of older people in Europe during the first months of the pandemic and to assess the combined role of age and gender.

**Methods:**

Wave 8 data of SHARE-corona (Survey of Health, Aging and Retirement in Europe) (n = 51,695, aged≥50) collected between Jun-Aug 2020 were used. Perceived health status was explored with a question on whether there has been a change compared with the health status before the COVID-19 outbreak (response options: worse, the same and better). Two-way ANOVA with interaction and Student's t-test with Bonferroni correction were used to compare the effects of gender and age group (50-59 years, 60-69 years, 70-79 years, and ≥80) in changes in perceived health.

**Results:**

Differences in perceived health were observed by age, as well as by gender in participants aged ≥70 years (F = 91.94; p < 0.001). These differences were significant both by gender (F = 19.39; p < 0.001) and age (F = 191.79; p < 0.001). No interaction was detected between both factors (p = 0.170), which allowed their effect to be studied individually. Among the people who reported a worsening in their perceived health, women aged 70 to 79 years predominated (11.1%), followed by men aged 80 and over (15.3%) and women of the same age group (16.4%).

**Conclusions:**

The results suggest an association between the change in perceived health during the pandemic and age. Women have a slightly worse health status than men in all age groups. Therefore, gender can be considered as an influential factor in perceived health in old age, which in turn can have a potential impact in the quality of life of older people.

**Funding:**

Projects Ref. H2019/HUM-5698 and Ref. 202010E158.

**Key messages:**

- Older people have been severely impacted by COVID-19 pandemic.
- The combined effect of age and gender on the change in perceived health status during the pandemic have been analyzed.

